# Supplementary material for: Accurate prediction of protein torsion angles using evolutionary signatures and recurrent neural network
Source: Sci Rep. 2021 Oct 26;11:21033. doi: 10.1038/s41598-021-00477-2 (PMC8548351; doi:10.1038/s41598-021-00477-2)
Supplement: Supplementary file 1 — Supplementary Information. [file 41598_2021_477_MOESM1_ESM.pdf]

Supplementary material
Accurate prediction of protein torsion angles using evolutionary signatures and recurrent neural network

Yong-Chang Xu, Tian-Jun ShangGuan, Xue-Ming Ding, Ngaam J. Cheung

The LSTM networks

In the ESIDEN, we use two types of LSTM networks including LSTM-1 and LSTM-2. In the LSTM-1 module, the final output h_t at the step t of the cell depends on other variables, including the input value $x_t = (x_1, x_2, ..., x_n) \in \mathbb{R}^n$, the forget gate $f_t \in \mathbb{R}^h$, the input gate $i_t \in \mathbb{R}^h$, the cell state $c_t \in \mathbb{R}^h$, the temporary cell state $\tilde{c}_t \in \mathbb{R}^h$ and the output gate $o_t \in \mathbb{R}^d$ at current state, and the cell state $c_{t-1} \in \mathbb{R}^h$ and the hidden values $h_{t-1} \in \mathbb{R}^h$ at the previous state, where the superscripts n and h are the numbers of input features and hidden units, respectively. The relationships among those variables are formulated as follows,

$$
\begin{aligned}
i_t &= \sigma(\mathbf{W}_{ix}x_t + \mathbf{W}_{ih}h_{t-1} + \mathbf{b}_i) \\
f_t &= \sigma(\mathbf{W}_{fx}x_t + \mathbf{W}_{fh}h_{t-1} + \mathbf{b}_f) \\
\tilde{c}_t &= \tanh(\mathbf{W}_{cx}x_t + W_{ht}h_{t-1} + \mathbf{b}_c) \\
c_t &= f_t \circ c_{t-1} + i_t \circ \tilde{c}_t \\
o_t &= \sigma_g(\mathbf{W}_o x_t + \mathbf{W}_{oh}h_{t-1} + \mathbf{b}_o) \\
h_t &= o_t \circ \tanh(c_t)
\end{aligned}
\tag{S1}
$$

where $\sigma(\cdot)$ is the Sigmoid function, and tanh is a nonlinear transformation function. $\mathbf{W}$ and $\mathbf{b}$ represent the weight matrix and bias vector of different activation units (e.g. $\mathbf{W}_{ix} \in \mathbb{R}^{d\times h}$ represent the input gate weight matrix), respectively. The operator $\circ$ denotes the Hadamard product.

In the ESIDEN, the LSTM-2 adopts an architecture of BiLSTM that is composed of the forward and backward LSTMs, which can capture previous and future context information. Based on the final forward and backward LSTM outputs, the LSTM-2 is expressed as follows,

$$
\begin{aligned}
h_t^F &= \sigma(\mathbf{W}_{Fx}x_t + \mathbf{W}_{FF}h_{t-1} + \mathbf{b}_F) \\
h_t^B &= \sigma(\mathbf{W}_{Bx}x_t + \mathbf{W}_{BB}h_{t-1} + \mathbf{b}_B) \\
h_t &= h_t^F \oplus h_t^B \\
y_t &= \mathbf{W}_{yF}h_t^F + \mathbf{W}_{yB}h_t^B + \mathbf{b}_y
\end{aligned}
\tag{S2}
$$

where $h_t^F \in \mathbb{R}^h$ and $h_t^B \in \mathbb{R}^h$ represent the forward and backward outputs. $\mathbf{W}$ and $\mathbf{b}$ represent the weight matrix and bias vectors, respectively. The $\oplus$ denotes the concatenate operating. For the LSTM-2, the units of each layer are 256, and the output of LSTM-2 doubles that of the LSTM-1.

Predicting structure with constraints

In the present study, we utilize an extended structure with only backbone atoms (N, C_α, C, O, and H) to represent a protein chain. Initially, the constraints of torsion angles and residue distances are collected and utilized to bias the sampling of MoDyFing (a module of *Leri*[1]). Once the set of final conformations in the first round is generate from MoDyFing simulations, the top 20% candidates with the lowest energy are chosen to generated structural information that can be merged and reused in the next round of simulations. In each round, MoDyFing moves on the space of torsion angles (ϕ, ψ) derived from the Ramachandran potential uses the NDRD TCB coil library[2], and by this way, it accelerates sampling with only two freedoms of each amino acid. Moreover, local folding dynamics can also be captured by the biased sampling in the space under the two constraints. In the previous round, the best-so-far structures are collected to compute the distribution of torsion angles (ϕ, ψ) that is to update sampling space and distances between pairwise residues that are to filter noises in the distance map. The dedicated information is reused to improve biased sampling the next round.

Dataset D2020

We download protein chains of 25% identity, resolution < 2.5 and R-factor < 0.25 from the PISCES server[3], totally 8,669 (as of December 2020). We filtered out protein whose sequence length less than 500 and obtained 7,443 proteins. The distribution of the protein size is illustrated in Figure S1. We randomly classify the chains into three groups with percentage ratio of 8 : 1 : 1, as shown in Table S1, 5,995 protein chains in the training dataset, 744 chains in the validation dataset, and the rest 744 chains for the test dataset.

Table S1. Summary of the D2020 dataset

Set	Training	Validation	Test	Total
Proteins	5,995	744	744	7,443

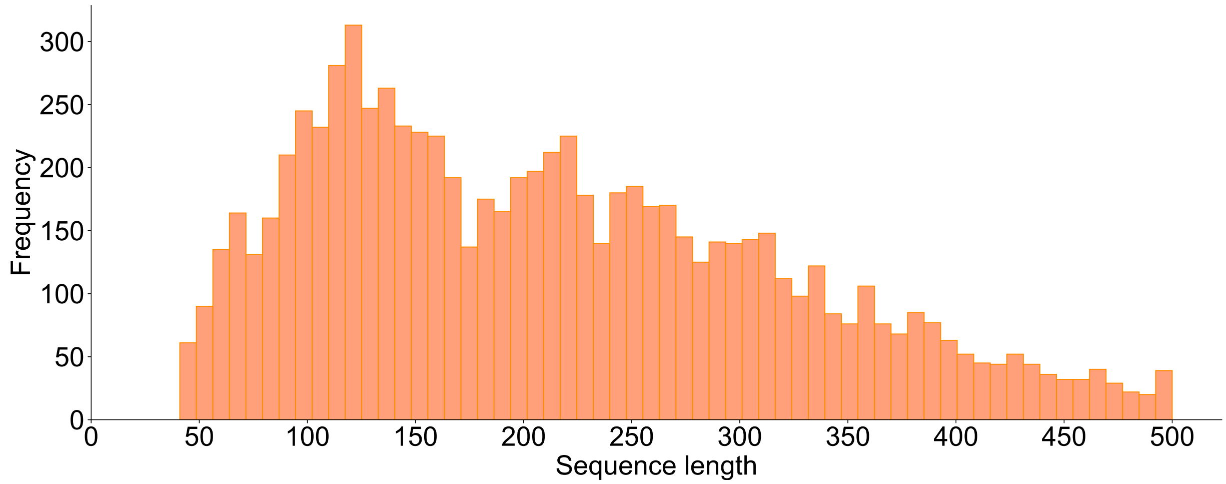

Figure S1. The distribution of the protein size on D2020 dataset.

Table S2. The MAE of combined features in predicting the torsion angles using ESIDEN on the D2020 dataset

Combined features	Validation		Test	
	MAE (ϕ)	MAE (ψ)	MAE (ϕ)	MAE (ψ)
Basic = PSSM + PP + AA	17.22 ± 0.08	25.19 ± 0.08	18.00 ± 0.08	25.87 ± 0.09
New = RE + DC + PSSP + RBP	16.64 ± 0.09	21.52 ± 0.16	17.24 ± 0.13	22.10 ± 0.09
Basic + DC	17.05 ± 0.06	24.88 ± 0.11	17.76 ± 0.08	25.33 ± 0.14
Basic + RE	17.18 ± 0.08	25.06 ± 0.08	17.82 ± 0.08	25.38 ± 0.09
Basic + PSSP	16.38 ± 0.07	23.21 ± 0.10	16.96 ± 0.08	23.51 ± 0.09
Basic + PSSP + DC	16.24 ± 0.02	23.21 ± 0.05	16.78 ± 0.02	23.50 ± 0.06
Basic + PSSP + RE	16.27 ± 0.03	23.20 ± 0.04	16.86 ± 0.04	23.41 ± 0.05
Basic + RBP	16.32 ± 0.06	21.08 ± 0.11	17.02 ± 0.09	21.70 ± 0.14
CbnF = Basic + DC + RE	17.01 ± 0.08	24.81 ± 0.09	17.64 ± 0.08	25.08 ± 0.08
CbnF + PSSP	16.21 ± 0.08	22.92 ± 0.09	16.78 ± 0.08	23.23 ± 0.07
CbnF + RBP	16.18 ± 0.09	20.89 ± 0.08	16.91 ± 0.09	21.59 ± 0.09
CbnF + PSSP + RBP	15.72 ± 0.07	20.06 ± 0.06	15.72 ± 0.07	19.77 ± 0.05

The SPOT-1D dataset

As shown in Table S3, chains of more than 700 residues are removed, and there are 10,029, 983, 1,213 proteins in train, validation and TEST2016, respectively. In addition, TEST2018 contains 250 proteins that are released between January 2018 and July 2018.

Table S3. Summary of the SPOT-1D dataset

Set	Training	Validation	TEST2016	TEST2018	Total
Proteins	10,029	983	1,213	250	12,475

CAMEO

We collected the recently released proteins released between March 2021 and June 2021 from CAEMO website (`https://www.cameo3d.org`). The protein with sequence length > 500 were removed, and we also removed proteins with $>25\%$ sequence identity using *needle*[4] against SPOT-1D dataset. Finally, the dataset consists of 109 proteins. As shown in Table S4, the MAE performance of ϕ predicted by the ESIDNE is better than Spider3 and RaptorX-Angle, and its MAE performance of ψ outperforms all the other three methods.

Table S4. Comparison among different methods on the CAMEO dataset

Methods	SPIDER3*	RaptorX-Angle*	SPOT-1D*	ESIDEN
MAE (ϕ)	17.89	19.57	16.49	16.57
MAE (ψ)	28.32	33.96	25.17	24.25

*The results are obtained locally using the Spider3, RaptorX-Angle, and SPOT-1D standalone packages, respectively

The CASP datasets

We collected the 59 template-free modeling targets (TFM) that have $<25\%$ sequence identity with SPOT-1D dataset from recent CASPs (`https://predictioncenter.org`), and we obtained 27, 11, 13, and 8 TFM proteins from the CASP11, CASP12, CASP13, and CASP14, respectively. The details of the 59 TFM targets are listed in Table S5.

As shown in Table S6, for comparison, the feature PSSP was extracted from different MSAs by using PSI-BLAST and HHblits. We compared the performance in terms of MAE on the feature PSSP from different MSAs, and the results show that the feature PSSP is not a compensate of PSSM but a new feature, as it is not heavily changed on different MSAs.

Table S5. Summary of the CASPs datasets

Targets	Residues	Targets	Residues	Targets	Residues	Targets	Residues
T0855-D1	119	T0794-D2	461	T0785-D1	108	T0791-D1	85
T0790-D1	257	T0761-D1	205	T0763-D1	129	T0781-D1	375
T0771-D1	155	T0808-D2	394	T0831-D2	363	T0767-D2	268
T0814-D1	390	T0834-D1	209	T0832-D1	205	T0802-D1	115
T0799-D1	401	T0775-D1	391	T0777-D1	343	T0789-D1	269
T0806-D1	255	T0793-D1	513	T0826-D1	536	T0810-D1	338
T0804-D1	194	T0824-D1	109	T0837-D1	128	T0900-D1	102
T0864-D1	236	T0886-D1	229	T0894-D1	142	T0869-D1	116
T0870-D1	312	T0859-D1	131	T0863-D1	582	T0878-D1	112
T0866-D1	112	T0914-D1	157	T0969-D1	254	T0960-D2	274
T0963-D2	364	T0957s1-D1	157	T0968s2-D1	116	T0986s2-D1	89
T0953s1-D1	72	T0980s1-D1	98	T0990-D1	552	T0987-D1	381
T1021s3-D1	149	T1022s1-D1	223	T1000-D2	450	T1046s1-D1	72
T1028-D1	125	T1082-D1	73	T1082-D1	134	T1038-D1	188
T1027-D1	168	T1064-D1	102	T1090-D1	189		

Table S6. The performance of PSSP derived from different MSAs on the CASP11, CASP12, CASP13, and CASP14

Tool for PSSP	CASP11 (27)		CASP12 (11)		CASP13 (13)		CASP14 (8)	
	MAE (ϕ)	MAE (ψ)	MAE (ϕ)	MAE (ψ)	MAE (ϕ)	MAE (ψ)	MAE (ϕ)	MAE (ψ)
PSI-BLAST	17.44	23.44	20.03	28.52	22.03	33.95	23.14	30.02
HHblits	17.25	23.30	19.94	28.86	22.15	32.40	23.01	29.96

Table S7. The TM-score and RMSD of the four inferred TFM targets by using predicted ϕ/ψ by Spider3, RaptorX-Angle, SPOT-1D and our method ESIDEN

Target	Spider3		RaptorX-angle		SPOT-1D		ESIDEN	
	TM-score	RMSD	TM-score	RMSD	TM-score	RMSD	TM-score	RMSD
T0986s1-D1	0.47	6.2	0.46	5.2	0.45	7.6	0.66	4.2
T0968s2-D1	0.53	5.6	0.53	6.6	0.53	6.7	0.72	4.8
T0957s1-D1	0.51	5.8	0.52	5.8	0.52	5.9	0.68	4.7
T0969-D1	0.78	5.4	0.76	4.8	0.77	4.7	0.77	6.8

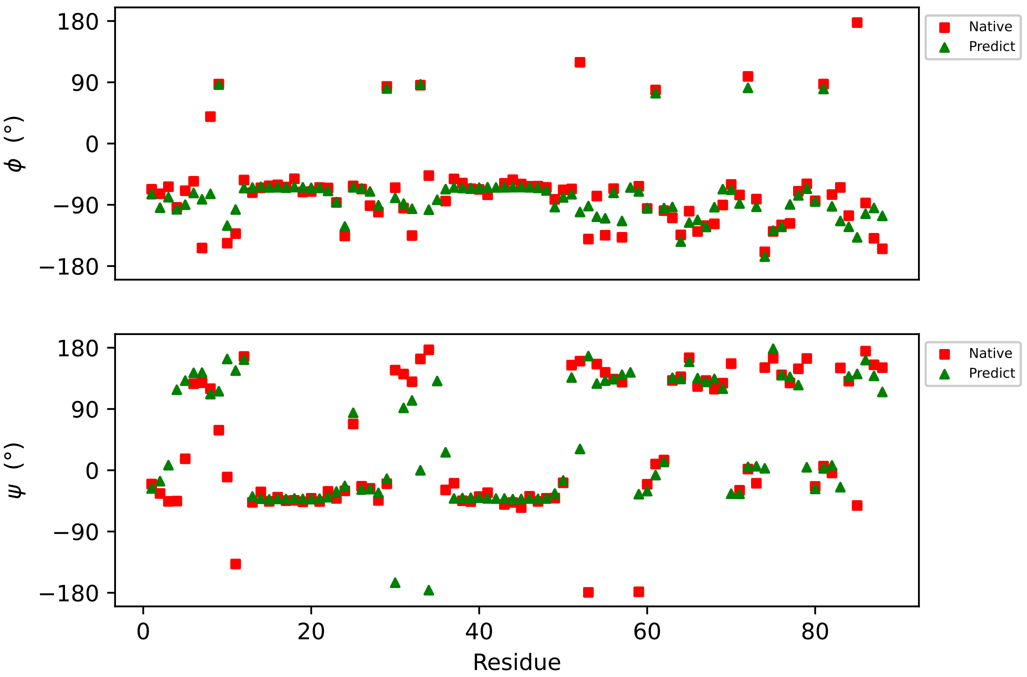

Figure S2. Comparison between the predicted and the native torsion angles of the TFM target T0968s2-D1

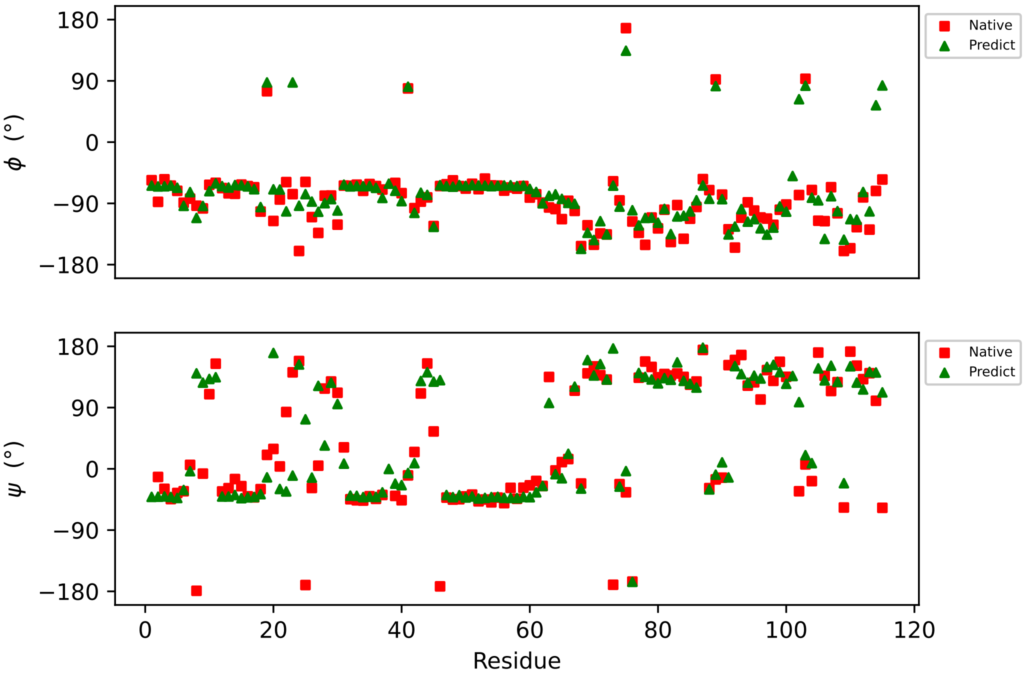

Figure S3. Comparison between the predicted and the native torsion angles of the TFM target T0986s1-D1

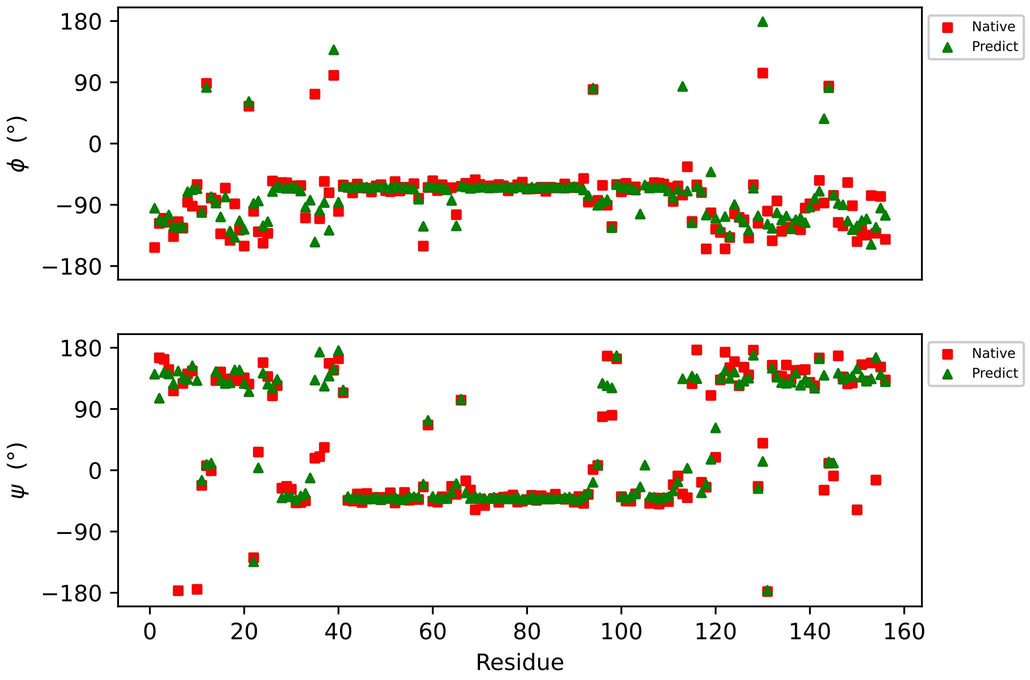

Figure S4. Comparison between the predicted and the native torsion angles of the TFM target T0957s1-D1

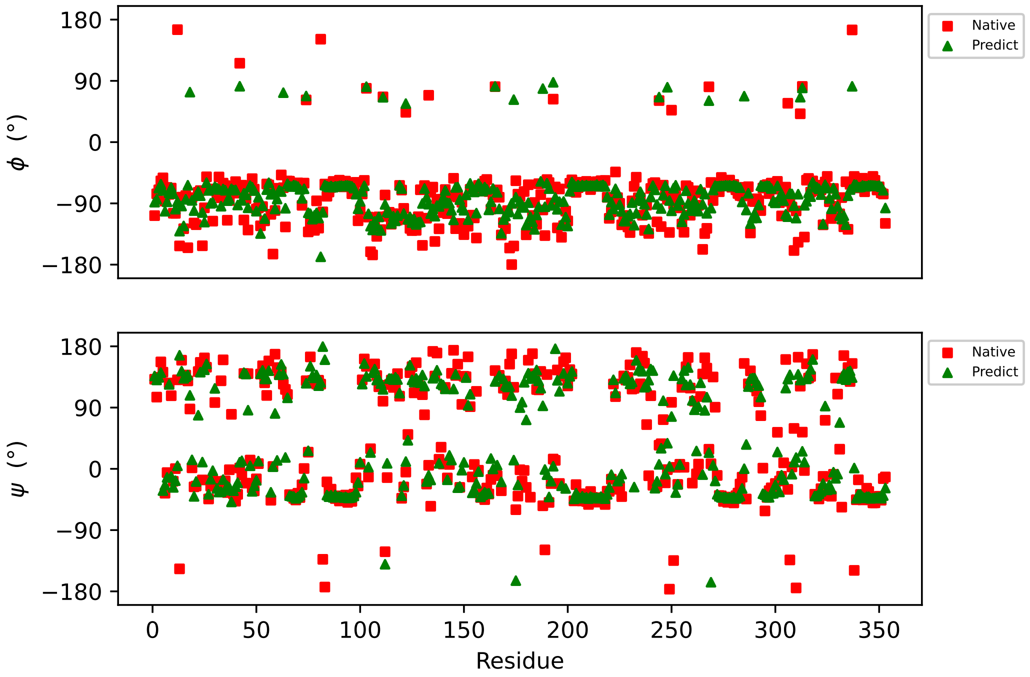

Figure S5. Comparison between the predicted and the native torsion angles of the TFM target T0969-D1

References

[1] Cheung, N. J., Peter, A. T. J. Kornmann, B. Leri: a web-server for identifying protein functional networks from evolutionary couplings. Computational and Structural Biotechnology Journal. 1–16 (2021).

[2] Ting, D.et al.Neighbor-dependent ramachandran probability distributions of amino acids developed from a hierarchical dirichlet process model. PLoS Comput. Biol 6, e1000763 (2010).

[3] Wang, G. Dunbrack, R. L. PISCES: recent improvements to a PDB sequence culling server. Nucleic acids research 33, W94–W98 (2005).

[4] Hancock J M , Bishop M J . EMBOSS (The European Molecular Biology Open Software Suite)[M]. John Wiley Sons, Inc. 2004.
